# Food insecurity and associated factors among adult HIV patients on anti-retroviral therapy in Dessie referral hospital, South Wollo Zone, North central Ethiopia

**DOI:** 10.1371/journal.pgph.0000445

**Published:** 2022-09-28

**Authors:** Amanuel Demisse, Melake Demena, Behailu Hawulte Ayele, Abrham Mengistu

**Affiliations:** 1 Public Health Expert at South Wollo Zone Guguftu Woreda Health Office, Guguftu, Ethiopia; 2 School of Public Health, College of Health and Medical Science, Haramaya University, Harar, Ethiopia; 3 Department of Public Health, College of Medicine and Health Science, Wechemo University, Hossana, Ethiopia; African Population and Health Research Center, KENYA

## Abstract

Food insecurity has a paramount negative impact on the overall nutritional and health status of people living with the human immune deficiency virus, hence leading to opportunistic infections, rapid disease progression, hospitalizations, poor treatment outcomes, and mortality, both are intertwined and worsen one another through a mixture of nutritional, mental health, and behavioral pathways that heighten vulnerability to, and worsen the severity of, each condition. Nevertheless, little is known about the magnitude of food insecurity and associated factors among adults on antiretroviral therapy in the current study area. This study aims to assess the magnitude of food insecurity and associated factors among Adults on Antiretroviral Treatment in Dessie referral hospital South Wollo Zone, Northcentral Ethiopia. An institution-based cross-sectional study was conducted among 407 selected adults living with Human Immune Virus receiving Anti-Retroviral treatment in Dessie referral hospital. Data was entered into Epi-data version 3.1 and exported to STATA version 16.0 for cleaning and analysis. Bivariable and multivariable binary logistic regression analysis was carried out to identify factors associated with the outcome variable. Odds ratio along with 95% confidence interval was estimated to measure the strength of the association and the level of statistical significance was declared at a p-value less than 0.05. This study revealed that the magnitude of food insecurity was 62.4% (95% CI: 57.6, 44 66.8]. CD4 count <350 [AOR = 3.51, 95% CI: 1.88, 6.52], average monthly household income ≤ 40 USD [AOR = 2.34, 95% CI: 1.42, 3.84], World Health Organization clinical stage III&IV [AOR = 2.85, 95% CI: 1.61, 5.04], not getting any support [AOR = 3.04, 95% CI: 1.45, 6.38] were factors significantly associated with food insecurity. Social protection interventions targeting patients with CD4 <350, monthly income less than 40 USD/month, World Health Organization clinical stage III &IV, and those patients with no support are crucial interventions for food security.

## Introduction

Food insecurity refers to the unavailability of adequate and sustainable food supply, inability to access an adequate balanced diet, and inability to utilize safe and quality food which is nutritionally adequate and socially acceptable ways for all household members [1]. According to Food and Agriculture Organization of the United Nations (FAO) reports it is estimated that over two billion people worldwide are affected by food insecurity that is linked to the HIV epidemic globally. The four building blocks of food security are availability, accessibility, utilization, and stability of foods [1–3].

The global prevalence of undernutrition was widely inconsistent with 10.8% worldwide, 19.9% Africa [4]. The Human Immune virus/Acquired Immunodeficiency syndromes (HIV/AIDS) remain global health problems that deteriorate household food insecurity by targeting the most productive and economic age group of the society. In sub- Saharan Africa around 28.5 million peoples are living with HIV/AIDS among 70% of them are food insecurity [5–9].

Food insecurity remains highly prevalent and has increasingly been recognized as a serious public health problem in developing countries [8]. The synergistic effect of both HIV infection and food insecurity harms the overall nutritional and health status of people living with HIV/AIDS leads to poor adherence to ART and upsurge transmission of HIV by increasing viral load and decreasing number CD4 count of patients hence leading to increased HIV-related opportunistic infections, poor clinical outcome, increased hospitalizations finally leads for mortality [6, 7].

Food insecurity and HIV/AIDS are intertwined and worsening one another in a vicious cycle through a mixture of nutritional, mental health, and behavioral pathways that heighten vulnerability to, and worsen the severity of, each condition. HIV infection by itself leads to food security and compromises nutritional status by reducing work capacity and productivity, and jeopardizing household livelihoods. In the community where there is High HIV prevalence, societies may face cumulative reductions in food supply and increased labor costs and extending the effect of interactions beyond the individual to the household and societal levels [7, 8, 10, 11].

Food insecurity can lead to both macronutrient and micronutrient deficiencies. These deficiencies affect the vertical and horizontal transmission of HIV that contributes to reduced immunity and lead to an increase in morbidity and mortality. It can have mental health consequences, including depression, increase drug abuse, accelerated HIV transmission, incomplete viral load suppression, and increased probability of AIDS-defining illness among HIV-infected persons [12]. Evidence indicates that small weight losses are associated with decreased survival rates due to reduced food intake, poor absorption of nutrients, and changes in the way the body uses nutrients [13].

The effect of food insecurity on HIV/AIDS and vice versa is known but little is known regarding the magnitude of food insecurity and associated factors among adults PLWHA on ART in resource-limited settings like Ethiopia. Therefore, this study aims to assess the magnitude of food insecurity and associated factors among people living with HIV in Dessie referral hospital in the north-central of Ethiopia.

## Methods

### Ethical statement

The study protocol was approved by the Haramaya University, College of Health and Medical Sciences Institutional Health Research Ethics Review Committee with reference no of IHRERC/180/2021. The permission and agreement consent was obtained from the Amhara Regional Health Bureau and Dessie referral hospital before the study. Informed, voluntary written and signed consent was obtained after the study participants were informed about the objective, procedure, benefit, and risk of the study. The full right and confidentiality of the participants were well maintained.

### Study setting

The study was conducted from June 25 –July 25, 2021, in Dessie Referral Hospital, South Wollo Zone, and North Central Ethiopia. Dessie is 500 km far from Bahir Dar, the capital of Amhara National Regional State, and 401km from Addis Ababa, the capital of Ethiopia. The city is found at a latitude and longitude of 11°8′N 39°38′E, with an elevation between 2,470 and 2,550 meters above sea level.The town has 5 health centers, 1 primary hospital, and 1 referral hospital. Dessie referral hospital has 28 health professionals working in the ART unit. Currently, a total of 6303 patients were actively attending the ART clinic at Dessie referral hospital, and 4870 of whom were adults above 18 years of age.

### Study design and population

An institutional-based quantitative cross-sectional study design was conducted among adults aged 18 years and above and attending anti-retroviral therapy (ART) at Dessie referral Hospital South Wollo Zone. Participants with severe illness, mentally sick, pregnant and lactating mothers, and those with incomplete patient files were excluded from the study.

### Sampling techniques and sample size determination

The maximum sample size of 420 was obtained considering the assumption for double population proportion formula (39.2 proportion of rural residents with food insecurity based on the study conducted in Kembata Tembaro Zone, Southern Ethiopia, [14]. an adjusted odds ratio of 1.3, 1:1 exposed to non-exposed ratio, 95% confidence interval, 80% study power, 5% margin of error, and 10% non-response rate) using Open Epi version 2.3.

A systematic random sampling technique was used to select the study participants. Accordingly, a serial of 12 intervals (4870 adult patients attending ART /420 sample size) was included using the patient registration number as a sampling frame. The first respondent was selected by the lottery method.

### Data collection tools and methods

The standard tool, Household Food Insecurity Access Scale (HFIAS) developed by Food and Nutrition Technical Assistant (FANTA) and United Nations Program on HIV/AIDS was used to collect data on the level of food insecurity. The tool has nine questions asking about three domains of food insecurity: feeling the uncertainty of food supply, insufficient quality of food, and insufficient food intake and its physical consequences [15]. Data on socio-demographic variables and clinical characteristics of the participants were collected by using structured and pretested questionnaires developed by reviewing different works of literature. Information regarding socio-demographic characteristics and food insecurity was collected using face-to-face interview techniques and data on the clinical characteristics of the participants were collected from the patient medical files. Anthropometric data were collected using a calibrated weight scale and a non-stretchable tap-meter following standard operative procedure.

### Measurement

Food Security Means when all respondents say “no” for all affirmative household food access scale of occurrence questions measured in terms of 9 items for at least four weeks (4) duration [15].

Food Insecure Individuals were labeled to be food insecure if they answer “Yes” to all affirmative household food access scale of occurrence questions measured in terms of 9 items for at least four weeks (4) duration. This can be labeled as mild, moderate, and severe food insecurity tertian classification method [15].

Mild Food Insecurity when respondents responded rarely (1) for frequency questions with a value interval between 1–9 inclusively [15].

Moderate Food Insecurity When respondents responded sometimes (2) for frequency questions with a value interval between 10–18 inclusively [15].

Severe Food Insecurity when respondents responded often (3) for frequency questions with a value of 27 (3X9 = 27) [15].

Dietary diversity is measured based on the number of reported different foods and food groups consumed in a household over 24 hours. This does not include food groups consumed outside the home. It is classified as adequate if the value is above the mean score and inadequate if it is below the mean score value depending on FANTA/FAO recommendations [16].

Body mass index was used to classify nutritional status: BMI<18.5, underweight; BMI 18.5–24.99, normal; and 25≤BMI >30, overweight and 30≤ BMI obese [16].

### Data quality control

The tool was first developed in the English language and translated to the local language, Amharic by the language experts, and back translation to English was made to check for consistency. The data were collected by qualified and experienced field staff (data collectors and supervisors). Data were collected by four trained diploma nurses and supervised by the research assistant (Master of Public Health graduates). A three days intensive training was given to the data collectors and the supervisors. The data collection instruments were pre-tested on 5% of ART clients attending the Kombolcha district hospital and some adjustment was made to the approaches of data collection and study tool. The anthropometric measurement was conducted according to the standard operating procedures. The supervisors and the research team have checked the data for completeness on an ongoing basis. Double entry was made to validate the entry and correct errors if any.

### Data processing and analysis

All filled questioners were checked for completeness and consistency and double data entry was done using the Epidata version.3.1 software. Then the data was exported to the STATA version 16.0 for cleaning and analysis. Descriptive statistics were used to present, frequency distribution, measure of central tendency, and measure of dispersion were used to describe the variables. Bivariable and multivariable logistic regression analysis was done to see the association between a dependent variable and explanatory variables. Accordingly, variables with a p-value of less than 0.25 during Bivariable logistic regression analysis were entered into a multivariable logistic regression model to control for all possible confounders. All the assumption of logistic regression (model adequacy and multicollinearity of the independent variable) was checked using appropriate methods. The absence of multi-collinearity was checked by using standard error <2. Model adequacy was checked by using the Hosmer and Lemeshow goodness of fit test having a P-value >0.05. Odds ratios along with a 95% confidence interval were estimated to measure the strength of the association. The level of statistical significance was measured at a P value less than 0.05.

## Result

### Socio-demographic characteristics

Out of 420 study participants, 407 have participated in the study making a response rate of 181 96.9%. More than half, 61.9% of the study participants were females and the mean (± SD) age of the participants was 35.21 (± 8.78). More than two-thirds 70% of the participants were married and 63.9% were rural residents. Around 92.6% of respondents’ families had less than five family sizes with a mean (±SD) family size were 3.6 (±1.3). The majority, 75.9% of the participants was Muslim by religion and 98.3% were Amhara ethnic group. Around two-fifth, 42% of the participants were farmers and 40.8% were illiterate (unable to write and read). The mean (±SD) income of the study participants was (1385(±1177) ETB (55.4 ±47.08 USD) (Table 1).

**Table 1:** Socio-demographic characteristics of adult people living with HIV on ART in Dessie referral hospital of South Wollo Zone, North-central Ethiopia 2021.

Variables		Frequency	Percent
Sex	Male	155	38.1
	Female	252	61.9
Age in years	18–25	59	14.5
	26–35	163	40
	36–44	111	27.3
	≥45	74	18.2
Residence	Urban	183	45
	Rural	224	55
Marital status	Single	58	14.3
	Married	285	70
	Divorced	32	7.9
	Widowed	32	7.9
Number of family members	≤ 5	377	92.6
	> 5	30	7.4
Educational status	Illiterate, unable to read and write	166	40.8
	Illiterate, able to read and write	68	16.7
	Primary (1–8)	80	19.7
	High school (9–12)	63	15.5
	Collage and above	29	7.1
Ethnicity	Amhara	400	98.3
	Tigrai	7	1.7
Religion	Protestant	17	4.2
	Orthodox	81	19.9
	Muslim	309	75.9
Occupational status	Farmer	171	42.0
	Merchant	31	7.6
	Government employer	25	6.1
	House wife	66	16.2
	Daily labor	98	24.1
	Student	16	3.9
Head of household	Male	224	55
	Female	183	45
Living condition	Alone	60	14.7
	With parents	182	44.7
	With relatives	22	5.4
	With spouse	143	35.1
Average monthly income	≤ 40 USD	238	58.5
	>40 USD	169	41.5

### Clinical characteristics

The mean (± SD) CD4 T-Lymphocyte cell count of the study participants was 476.50 (±235.33). More than one-fifth, 21.6% of the participants have a CD4 count >500 cells/mm3. The majority of the study participants, 86% were in WHO clinical stage I&II and 77.2% were on a 1J (TDF+3TC+DTG) treatment regimen. Among the total participants, 56.3% of participants have HIV-positive family members with a spouse the most reported HIV-positive family members. The mean duration of participants on ART was 90.43 (±48.97) months. Most of the respondents, 92.86% had working functional status and the ability to perform usual work inside or outside the home. Around two-fifths of the participants had an opportunistic infection/s of any kind in the last six months and the most reported opportunistic infection was pneumonia. The majority of respondents 86.5% did not have any support from governmental or non-governmental organizations (Table 2).

**Table 2:** Health-related and immunological profiles of ART patients in Dessie referral Hospital of South Wollo Zone, North-central Ethiopia, June25-July25, 2021.

Variables	Category		Frequency	Percentage
CD4+ T cell count	<350		251	61.7
	350–500		68	16.7
	>500		88	21.6
WHO clinical stage	Stage I& II		315	77.2
	Stage III&IV		92	22.5
Duration on ART	<12 months		39	9.6
	≥12 months		368	90.4
ART regimens	1J(TDF+3TC+DTG)		311	76.4
	1E(TDF+3TC+EFV)		81	19.9
	2H(TDF+3TC+ATV/r)		15	3.7
Developing OIs in the past six months	No		249	61.2
	Yes	Disease	158	38.8
		TB	17	17.9
		Pneumonia	110	69.6
		Oral thrush	10	10.5
		Zoster	3	3.2
		Diarrhea	36	37.9
Any support from governmental or non-governmental organization	No		352	86.5
	Yes Support		55	13.5
		Money	28	60
		Food	16	29
		Loan	36	65
		Equipment	4	7
Other person living with HIV on ART in the family	No		178	43.7
	Yes	Persons	229	56.3
		Spouse	191	83.4
		Child	36	15.7
		Parents	26	11.4
		Other relatives	3	1.3
Functional status	Working		377	92.6
	Ambulatory		30	7.4
BMI	<18.5Kg/m^2^		80	19.7
	18.5Kg/m^2^-24.9Kg/m^2^		293	72.0
	≥25Kg/m^2^		34	8.3
Dietary Diversity	Adequate		197	48.4
	Inadequate		210	51.6

### Nutritional status of adults on ART

Among the study participants, 19.7% had BMI < 18.5 (underweight), and 72% were in the normal range. Among the respondents, 33.2% reported their actual daily meal pattern to be less than 3 times which is below the daily recommended meal frequency for PLHIV on ART. Among study participant’s 44.2%, 30%, and 57.7% escape breakfast, lunch, and dinner respectively. Dietary diversity score was assessed using 9 food items dietary diversity scale in the study group. More than half, 51.6% of the study participants have inadequate dietary diversity. Other vitamin rich fruits and vegetables were consumed by 82.4% of respondents. The primary source food for 57.7% of the respondents was own production.

### Health-related and immunologic factors among adults on ART

The median CD4 T-Lymphocyte cell count of the participants was 428 with the range of 218 to 1307. Almost one-fifth, 21.6% of the participants were with CD4 count >500. The majority of the study participants, 86% were in WHO clinical stage I&II and 77.2% were on a 1J (TDF+3TC+DTG) treatment regimen. Among the total participants, 56.3% of participants have HIV-positive family members and spouses were the most reported HIV-positive family members. The median duration of participants on ART was 84 months with a minimum of 3 months and a maximum of 204 months and a range of 201. Most of the respondents, 92.86% had working functional status and the ability to perform usual work inside or outside the home. Around two-fifths of the participants had an opportunistic infection/s of any kind in the last six months and the most reported opportunistic infection was pneumonia. The majority of respondents 86.5% did not have any support from governmental or non-governmental organizations (Table 2).

### The magnitude of food insecurity among adults on ART

The overall magnitude of food insecurity in this study was 62.4% (95% CI: 57.6, 66.8). Of which 74(18.1%), 95% CI: (16.7–20.8) were mildly food insecure 96 (23.5%), 95% CI: (21.1–25.2) were moderately food insecure 83 (20.4%), 95% CI: (18.3–22.7) were severely food insecure and 158(37.6.4%) 95% CI: (30.5–40.8) were food secured.

### Factors associated with food insecurity among adults on ART

All variables with a P-value of ≤ 0.25 in bivariable analysis were included in the multivariable regression model. In multivariable analysis, CD4count <350, being WHO stage III&IV, absence of any support, and Low average monthly income were factors associated with food insecurity among adults attending ART. Participants with CD4 count <350 were 3.51 times more likely to have food insecurity than those with CD4 count ≥350 [AOR = 3.51, 95% CI (1.88–6.52)]. Participants with the average monthly household income of ≤ 1000 ETB were 2.34 times more likely to be food insecure than those with an income >1000ETB [AOR: 2.34, 95%CI (1.42–3.84)]. Those with advanced WHO clinical stage (III&IV) were 2.85 times more likely to have food insecurity than stage I&II participants [AOR = 2.85, 95% CI (1.61–5.04)]. Those participants who did not get any support were 3.04 times more likely food insecure than those who get support [AOR: 3.04, 95% CI (1.45–6.38)] (Table 3).

**Table 3:** Multivariable logistic regression analysis of factors associated with food insecurity in Dessie referral hospital of South Wollo Zone, North-central Ethiopia 2021.

Variables	Category	Food security status		COR	AOR
		Insecure	Secure	(95%CI)	(95%CI)
Sex	Male	115(28.2%)	40(9.8%)	1	1
	Female	138(34%)	114(28%)	0.42(0.39–1.12)	0.87(0.50–1.53)
Residence	Urban	111(27.2%)	72(17.7%)	1	1
	Rural	142(34.8%)	82(20.1%)	1.20(0.51–2.16)	0.77(0.45–1.32)
Head of household	Male	142(34.8%)	82(20.1%)	1	1
	Female	111(27.2%)	72(17.6%)	1.12(0.47–3.07)	0.71(0.40–1.27)
Monthly income	≤1000ETB	216(53%)	22(5.4%)	35.02(19.04–45.40)	**2.34(1.42–3.84)** *
	>1000ETB	37(9.1%)	132(32.4%)	1	1
WHO stage of disease	Stage I&II	169(41.5%)	146(35.8%)	1	1
	Stage III& IV	84(20.6%)	8(1.9%)	9.07(5.92–35.00)	**2.85(1.61–5.04)** *
Developed OIs in the last 6 months	Yes	69(16.9%)	89(21.8%)	3.65(2.01–8.29)	1.24(0.74–2.07)
	No	184(45.2%)	65(15.9%)	1	1
Support	Yes	23(5.6%)	32(7.8%)	1	1
	No	230(56.5%)	122(29.9%)	2.62(1.83–3.88)	**3.04(1.45–6.38)** *
CD4+ T cell count	≤350	190(46.7%)	61(15.1%)	4.94(2.07–8.53)	**3.51(1.88–6.52)** *
	350–500	29(7.1%)	39(9.5%)	1.18(0.45–6.31)	0.91(0.39–2.10)
	≥500	34(8.3%)	54(13.2%)	1	1
Dietary diversity	Inadequate	120(29.4%)	90(22.1%)	1.55(1.45–2.02)	0.65(0.38–1.09)
	Adequate	133(32.6%)	64(15.7%)	1	1

* Significant at P-value <0.05, COR = Crude Odd Ratio, AOR = Adjusted odd ratio, CI = Confidence interval, OIs = opportunistic infections.

## Discussion

The current study revealed the magnitude of food insecurity among PLWHA was found to be 62.4%. Low CD4+ T cell count, WHO stage of III&IV, absence of any support, and monthly income were factors that were significantly associated with food insecurity. The magnitude was higher than studies conducted in west shewa 19.5% [10], Kembata 57.3% [14], A/minch 35.2% [9], and study conducted in West Benga 50.9% [17] and lower than study conducted in Debre Markos 84.5% [18], Hawassa Referral Hospital 67.3% [19] Zambia 74% [20], Dakar, Senegal 84.6% [21], Congo 91.3% [22] and consistent with the study done in Brazil 66.5% [16]. The variation could be due to the existence of different socioeconomic statuses, the health intervention measurement is taken, the difference in study years, and study setting [23].

This study showed that CD4 was strongly associated with food insecurity. Respondents who had CD4 count <350 were more likely to be food insecure than those who had CD4 count ≥350. This result is supported by a study done in west Shewa [13]. Evidence indicated that food insecurity was associated with lower CD4 count and it tends to decrease CD4 counts recovery and predisposes patients to early death [13].

This study revealed that monthly income was strongly associated with food insecurity. Participants who had low monthly income (≤1000 ETB) were more likely to be food insecure than those who had >1000 ETB. This finding is in accordance with the study conducted at Arbaminch [9], Debere markos [18], Kembata [14], Senegal [21], Zambia [20], Brazil [16].

In this study low income significantly increased the likelihood of food insecurity among adults on ART drugs because low income or lacked income is not able to afford adequate food and therefore are food insecure. When income diminishes in a household may cause inadequate quality and quantity of food intake due to unable to purchase variety and preferences of the type of food, anxiety, and uncertainty about the household food supply [18]. Moreover, it will cause the individuals to reduce dietary energy to intakes below daily requirements [24]. Food insecurity and low income found among adults living with HIV on ART drugs in this study could also be a result of HIV/ AIDS. This is because frequent illnesses including opportunistic infections cause significant disability leading to reduced productivity, depletion of savings, and inability to earn more incomes this indicates that HIV/AIDS could lead to food insecurity through diminished earning of an individual [18].

This study revealed that participants who are on WHO stage III and IV were more likely to have food insecurity as compared to WHO stage I and II. This finding is supported by research conducted in Arbaminch [9], Kembata [14]. As WHO staging increases the patient becomes physically weak and CD4 count decreased it resulting to be less productive. As indicated in different studies food insecurity has been associated with a range of adverse clinical effects among PLWHA, including declines in physical health status, decreased viral suppression increased incidence of serious illness, and end up in food insecurity [9].

This study revealed that the absence of any support was significantly associated with food insecurity. Participants those not have any type of support (food, money, loan, or livestock) were more likely food insecure than those who have supported this finding is supported by a study done in kembata [14], Congo [22]. This could be due to those who got support from the government or other nongovernmental organizations being less likely to be food insecure. In addition, food and nutrition support results in increased immune system strength, and this makes persons more productive and earns more money [14]. The findings of this study should be interpreted with some limitations. Respondents may not tell the real information about their food security status due to the need for aid.

This study had the following limitations; the study used participant interviews about events in the past one month there could be recall bias by participants and also there may be social desirability bias. This was minimized by probing the respondents about the event.

## Conclusions

The magnitude of food insecurity among adult ART attendants was 62.4% of which 18.1% mildly, 23.5% moderately and 20.4% were severely food insecure. CD4count <350, being WHO stage III&IV, absence of any support, and Low average monthly income were factors associated with food insecurity among adults attending ART. We recommended applying the national nutrition program component of food and nutrition interventions integration with HIV/AIDS care, treatment, and support and also linking of food insecure individuals to the national productive safety net program.

## Supporting information

S1 Text Questionnaire used to assess food insecurity and associated factors among Adult HIV patients on anti-retroviral. (DOCX)

S1 Data Raw data used in the analysis of food insecurity and associated factors among adult HIV patients on anti-retroviral. (SAV)

## References

[1] Carlson SJ, Andrews MS, Bickel GW. Measuring food insecurity and hunger in the United States: development of a national benchmark measure and prevalence estimates. J Nutr. 1999: 510S–516S. doi: 10.1093/jn/129.2.510S . 10064320

[2] Bhalla G., Handa S., Angeles G., and Seidenfeld D., “The effect of cash transfers and household vulnerability on food insecurity in Zimbabwe,” in Innocent Working Paper UNICEF Office of Research, vol. 22, no. 1, pp. 37–42, UNICEF office of research, Florence, 2016 Available at https://www.unicef-irc.org/publications/pdf/IWP_2016_22.

[3] World Bank, “Global Economic Prospects, January: Darkening Skies. Washington, DC; International Monetary Fund (IMF),” in World Economic Outlook, April 2019: Growth Slowdown, Precarious Recovery IMF, Washington, DC; International Monetary Fund (IMF).

[4] McGuire S. WHO, World Food Programme, and International Fund for Agricultural Development. 2012. The State of Food Insecurity in the World 2012. Economic growth is necessary but not sufficient to accelerate reduction of hunger and malnutrition. Rome, FAO. Adv Nutr. 2013 Jan 1;4(1):126–7. doi: 10.3945/an.112.003343 ; PMCID: PMC3648734. 23319131 PMC3648734

[5] FAO, IFAD, UNICEF, WFP and WHO, “The State of Food Security and Nutrition in the World,” in Safeguarding against economic slowdowns and downturns, FAO, Rome, 2019 Available at https://www.fao.org/3/ca5162en/ca5162en.

[6] World Bank: Poverty and Hunger: Issues and options for food security in developing countries. Washington. USA: World Bank; 1986 Available at https://documents1.worldbank.org/curated/en/166331467990005748/pdf/multi-page.

[7] Devereux S, Sussex I. Food insecurity in Ethiopia. In: A DFID Ethiopia Seminar, London; 2000 Available at https://www.ids.ac.uk/download.php?fil=files/FoodSecEthiopia4e.

[8] Frega R, Duffy F, Rawat R, Grede N. Food Insecurity in the Context of HIV/AIDS: A Framework for a New Era of Programming. Food and Nutrition Bulletin. 2010;31(4_suppl4):S292–S312. doi: 10.1177/15648265100314S402 21214035

[9] Mensa M, and Zelalem NB. “Levels and Predictors of Food Insecurity among HIV Positive Adult Patients Taking Highly Active Anti-Retroviral Therapy at Arba Minch General Hospital, Southern Ethiopia, 2016.” *Genetics in Medicine* 5 (2017): 1–11.

[10] Oluma A, Abadiga M, Mosisa G, Etafa W, Fekadu G. Food Insecurity among People Living with HIV/AIDS on ART Follower at Public Hospitals of Western Ethiopia. International Journal of Food Science. 2020;2020:8825453. doi: 10.1155/2020/8825453 32802831 PMC7416256

[11] Weldegebreal F, Digaffe T, Mesfin F, Mitiku H. Dietary diversity and associated factors among HIV positive adults attending antiretroviral therapy clinics at Hiwot Fana and Dilchora Hospitals, eastern Ethiopia. *HIV AIDS (Auckl)*. 2018;10:63–72 doi: 10.2147/HIV.S138638 29861644 PMC5968811

[12] Palermo T, Rawat R, Weiser SD, Kadiyala S (2013) Food Access and Diet Quality Are Associated with Quality of Life Outcomes among HIV-Infected Individuals in Uganda. PLOS ONE 8(4): e62353. doi: 10.1371/journal.pone.0062353 23638049 PMC3630150

[13] Gebremichael DY, Hadush KT, Kebede EM, Zegeye RT. Food Insecurity, Nutritional Status, and Factors Associated with Malnutrition among People Living with HIV/AIDS Attending Antiretroviral Therapy at Public Health Facilities in West Shewa Zone, Central Ethiopia. Biomed Res Int. 2018 May 6;2018:1913534. doi: 10.1155/2018/1913534 ; PMCID: PMC5960526. 29854730 PMC5960526

[14] Markos M., Egata G., and Dessie Y., Food Insecurity and Associated Factors among Adult HIV Positives Attending Antiretroviral Therapy in Public Health Facilities of Kembata Tembaro Zone, Southern Ethiopia. East African Journal of Health and Biomedical Sciences 2018. 2: p. 23–24.

[15] FAO, Guideline for measuring household and individual dietary diversity Prepared by FAO Nutrition and Consumer Protection Division with support from the EC/FAO Food Security Information for Action Programme and the Food and Nutrition Technical Assistance. 2008 Available at https://www.fao.org/publications/card/en/c/5aacbe39-068f-513b-b17d-1d92959654ea/

[16] Yang J., Qiu H., Li H., Zhang Y., Tao X. and Fan Y. (2015) Body Mass Index, Waist Circumference and Cut-Off Points for Metabolic Syndrome in Urban Residents in Ningxia. *Open Journal of Endocrine and Metabolic Diseases*, 5, 163–170. doi: 10.4236/ojemd.2015.512020

[17] Dasgupta P, Bhattacherjee S, Das DK. Food Security in Households of People Living With Human Immunodeficiency Virus/Acquired Immunodeficiency Syndrome: A Cross-sectional Study in a Subdivision of Darjeeling District, West Bengal. J Prev Med Public Health. 2016 Jul;49(4):240–8. doi: 10.3961/jpmph.16.023 ; PMCID: PMC4977769. 27499166 PMC4977769

[18] Chanie W, Negessie A, Dagnew Z (2017) Magnitude of Food Insecurity and Associated Factors among Adult Individuals on Anti-Retroviral Drug at Debre Markos Referral Hospital, Northwest Ethiopia, 2017: Cross-Sectional Study Design. Food Nutr J 2: 155. doi: 10.29011/2575-7091.100055

[19] Rekiku F and Tamirayehu SH, Household food security and associated factors among adult people living with HIV/AIDS attending ART clinic in Hospitals of Hawassa Town, Southern Ethiopia Research square, 2018 10.21203/rs.2.14384/v1

[20] Masa R, Chowa G, Nyirenda V. Prevalence and Predictors of Food Insecurity among People Living with HIV Enrolled in Antiretroviral Therapy and Livelihood Programs in Two Rural Zambian Hospitals. Ecol Food Nutr. 2017 May-Jun;56(3):256–276. doi: 10.1080/03670244.2017.1311256 Epub 2017 Apr 18. ; PMCID: PMC5953554. 28418728 PMC5953554

[21] Benzekri NA, Sambou J, Diaw B, Sall EHI, Sall F, Niang A, et al. (2015) High Prevalence of Severe Food Insecurity and Malnutrition among HIV-Infected Adults in Senegal, West Africa. PLOS ONE 10(11): e0141819. doi: 10.1371/journal.pone.0141819 26529509 PMC4631507

[22] Akilimali PZ, Musumari PM, Kashala-Abotnes E, Tugirimana PL, Mutombo PB, Veldman JF, et al. (2016) Food insecurity and Page 3 of 9 undernutrition in treated HIV patients a (post-) conflict setting: A cross sectional study from Goma, Eastern Democratic Republic of Congo. J Nutrition Health Food Sci 4(2): 1–9. 10.15226/jnhfs.2015.00163

[23] Medeiros ARC, Lima RLFC, Medeiros LB, Trajano FMP, Salerno AAP, Moraes RM, et al. Moderate and severe household food insecurity in families of people living with HIV/Aids: scale validation and associated factors. Cien Saude Colet. 2017 Oct;22(10):3353–3364. Portuguese, English. doi: 10.1590/1413-812320172210.02462017 . 29069190

[24] Asnakew M. (2015) Food Insecurity: Prevalence and Associated Factors among Adult Individuals Receiving Highly Active Antiretroviral Therapy (HAART) in ART Clinics of Hosanna Town, Hadiya Zone, Southern Ethiopia. *Open Access Library Journal*, 2, 1–9. doi: 10.4236/oalib.1101800

