# Supplementary material for: Food insecurity and associated factors among adult HIV patients on anti-retroviral therapy in Dessie referral hospital, South Wollo Zone, North central Ethiopia
Source: PLOS Glob Public Health. 2022 Sep 28;2(9):e0000445. doi: 10.1371/journal.pgph.0000445 (PMC10021340; doi:10.1371/journal.pgph.0000445)
Supplement: S1 Text — (DOCX) [file pgph.0000445.s001.docx]

Questionnaire No.: ___/___/___/ ART Center/site: _______________

Date of the questionnaire filled (dd/mm/yy): ____/__/___

Start Time: ____/____/: end Time____/____/

**Part I: Socio-Demographic Characteristics**

| **No** | **Question** | **Response** | **Code** | **Skip** |
| --- | --- | --- | --- | --- |
| 101 | Sex of respondent | 1. Male 2. Female |  |  |
| 102 | Age of respondents in year | ______________years |  |  |
| 103 | Place of Residence | 1. Urban 2. Rural |  |  |
| 104 | Current Marital Status | 1. Single 2. Married 3. Divorced 4. widowed |  |  |
| 105 | How many family members are there including you? |  |  |  |
| 106 | Dou you have children? | 1. Yes 2. No |  |  |
| 107 | If yes to Q106 how many children do you have? |  |  |  |
| 108 | Educational status | 1. have no formal education 2. Read and write 3. Grade( 1-4) 4. Grade( 5-8) 5. high school( 9 -12) 6. college and above |  |  |
| 109 | Ethnicity of respondent | 1. Amhara 2. Tigre 3. Afar 4. Oromo   5 Others(specify) |  |  |
| 110 | Religion of Respondent | 1. Protestant 2. Orthodox 3. Catholic 4. Muslim   5 Others, specify_ |  |  |
| 112 | Current Occupation | 1. Farmer 2. Merchant 3. Government Employee 4. House wife 5. Daily Laborer 6. Student   7 Others(specify |  |  |
| 113 | Who is the head of household? | 1. Male 2. Female |  |  |
| 114 | Living condition | 1. Alone 2. With parents 3. With relatives 4. With spouse 5. Others (specify) |  |  |
| 115 | Average monthly house hold income for the family | ________________ETB |  |  |
| 116 | Where is you or your households the primary source for obtaining food | 1. Own production 2. Purchased 3. Borrowed, gift 4. Food aid 5. Other (specify)… |  |  |

**Part II: Dietary Diversity and meal frequency related questions**

| Now I would like to ask you about the types of foods that you ate yesterday during the day and at night | | | code |  |
| --- | --- | --- | --- | --- |
| 201 | Any maize, teff, rice, wheat, sorghum, millet or any other grains or foods made from these (e.g. Injera, kita, qollo, bread, noodles, porridge or other grain products) | 1 Yes  2 No |  |  |
| 202 | Any pumpkin, carrot, sweat potatoes, red sweet pepper? | 1 Yes  2 No |  |  |
| 203 | Any white potatoes, white yams, or other foods made from roots | 1 Yes  2 No |  |  |
| 204 | Any dark green/leafy vegetables, including wild ones + locally available vitamin-A rich leaves such as amaranth, kale, spinach, green pepper | 1 Yes  2 No |  |  |
| 205 | Any other vegetables (e.g. tomato, onion, ) | 1 Yes  2 No |  |  |
| 206 | Any ripe mangoes, cantaloupe, ripe papaya, dried peaches | 1 Yes  2 No |  |  |
| 207 | Any fruits? (Mango, Papaya, Orange etc.) | 1 Yes  2 No |  |  |
| 208 | Any liver, kidney, heart or other organ meats or blood-based foods | 1 Yes  2 No |  |  |
| 209 | Any beef, pork, lamb, goat, rabbit, game, chicken, duck, other birds, insects meat | 1 Yes  2 No |  |  |
| 210 | Any eggs? | 1 Yes  2 No |  |  |
| 211 | Any fresh or dried fish or shellfish? | 1 Yes  2 No |  |  |
| 212 | Any foods made from beans, peas, or lentils? | 1 Yes  2 No |  |  |
| 213 | Any cheese, yogurt, milk or other milk products? | 1 Yes  2 No |  |  |
| 214 | Any foods made with oil, fat, or butter? | 1 Yes  2 No |  |  |
| 215 | Any sugar, sweetened soda or sugar foods such as chocolate, sweets/candies | 1 Yes  2 No |  |  |
| 216 | Any other foods, such as condiments, coffee, tea? | 1 Yes  2 No |  |  |

| During the previous 24-hours period (yesterday day and night), did you or anyone in your  household consume | | | | |
| --- | --- | --- | --- | --- |
| 217 | Any food Before a morning meal | 1 Yes  2 No |  |  |
| 218 | Morning meal | 1 Yes  2 No |  |  |
| 219 | Any food between morning and midday meals | 1 Yes  2 No |  |  |
| 220 | A midday meal | 1 Yes  2 No |  |  |
| 221 | Any food between midday and evening meal | 1 Yes  2 No |  |  |
| 222 | Any evening meal | 1 Yes  2 No |  |  |
| 223 | Any food after the evening meal | 1 Yes  2 No |  |  |

**Part III. Food security Condition (HFIAS) related questions**

| 301 | In the past four weeks, did you worry that your household would not have enough food? | | 0 No  1 Yes |  | Q302 |
| --- | --- | --- | --- | --- | --- |
| 301a | How often did this happen? | 1 Rarely (Once or twice in the past four weeks)  2 Sometimes (3 to 10 times in the past four weeks)  3 Often (more than 10 times in the past four weeks) | 1  2  3 |  |  |
| 302 | In the past four weeks, were you or any household member not able to eat the kinds of foods you/he/she preferred because of a lack of resources? | | 0 No  1 Yes |  | Q303 |
| 302a | How often did this happen? | 1 Rarely (Once or twice in the past four weeks)  2 Sometimes (3 to 10 times in the past four weeks)  3 Often (more than 10 times in the past four weeks) | 1  2  3 |  |  |
| 303 | In the past four weeks, did you or any household member have to eat a limited variety of foods due to a lack of resources? | | 0 No  1 Yes |  | Q304 |
| 303a | How often did this happen? | 1 Rarely (Once or twice in the past four weeks)  2 Sometimes (3 to 10 times in the past four weeks)  3 Often (more than 10 times in the past four weeks) | 1  2  3 |  |  |
| 304 | In the past four weeks, did you or any household member have to eat some foods that you really did not want to eat because of a lack of resources to obtain other types of food? | | 0 No  1 Yes |  | Q305 |
| 304a | How often did this happen? | 1 Rarely (Once or twice in the past four weeks)  2 Sometimes (3 to 10 times in the past four weeks)  3 Often (more than 10 times in the past four weeks) | 1  2  3 |  |  |
| 305 | In the past four weeks, did you or any household member have to eat a smaller meal than you felt you needed because there was not enough food? | | 0 No  1 Yes |  | Q306 |
| 305a | How often did this happen? | 1 Rarely (Once or twice in the past four weeks)  2 Sometimes (3 to 10 times in the past four weeks)  3 Often (more than 10 times in the past four weeks) | 1  2  3 |  |  |
| 306 | In the past four weeks, did you or any other household member have to eat fewer meals in a day because there was not enough food? | | 0 No  1 Yes |  | Q307 |
| 306a | How often did this happen? | 1 Rarely (Once or twice in the past four weeks)  2 Sometimes (3 to 10 times in the past four weeks)  3 Often (more than 10 times in the past four weeks) | 1  2  3 |  |  |
| 307 | In the past four weeks, was there ever no food to eat of any kind in your household because of lack of resources to get food? | | 0 No  1 Yes |  | Q308 |
| 307a | How often did this happen? | 1 Rarely (Once or twice in the past four weeks)  2 Sometimes (3 to 10 times in the past four weeks)  3 Often (more than 10 times in the past four weeks) | 1  2  3 |  |  |
| 308 | In the past four weeks, did you or any household member go to sleep at night hungry because there was not enough food? | | 0 No  1 Yes |  | Q309 |
| 308a | How often did this happen? | 1 Rarely (Once or twice in the past four weeks)  2 Sometimes (3 to 10 times in the past four weeks)  3 Often (more than 10 times in the past four weeks) | 1  2  3 |  |  |
| 309 | In the past four weeks, did you or any household member go a whole day and night without eating anything because there was not enough food? | | 0 No  1 Yes |  |  |
| 309a | How often did this happen? | 1 Rarely (Once or twice in the past four weeks)  2 Sometimes (3 to 10 times in the past four weeks)  3 Often (more than 10 times in the past four weeks) | 1  2  3 |  |  |

**Part IV Coping Strategies related questions**

These are asked for those who gave response YES for ANY of the above questions of Food security Condition (HAFIS) questions.

| 401 | What did you do when you or one of your household comes up with shortage of food during the past four weeks? |  | **Yes** | **No** |
| --- | --- | --- | --- | --- |
|  |  | Rely on less preferred foods | 1 | 2 |
|  |  | Purchase food on credit | 1 | 2 |
|  |  | Receiving food from food relaive, church, friends | 1 | 2 |
|  |  | Consume seed stock held for next season | 1 | 2 |
|  |  | Send children to eat with neighbors | 1 | 2 |
|  |  | Send household members to beg | 1 | 2 |
|  |  | Limit portion size at meal times | 1 | 2 |
|  |  | Restrict consumption by adults | 1 | 2 |
|  |  | Ration the money you have and buy prepared food | 1 | 2 |
|  |  | Reduce number of meals eaten in a day | 1 | 2 |
|  |  | Sex for money or food | 1 | 2 |
|  |  | Skip entire days without eating | 1 | 2 |
|  |  | Selling essential assets like land, house, ox | 1 | 2 |

**Part V. Disease related and immunologic status related question**

| 501 | The last CD4 Level count | ______________ cell/dl |  |
| --- | --- | --- | --- |
| 502 | WHO clinical stage | 1 stage I  2 stage II  3 stage III  4 stage IV |  |
| 503 | For how long have you been taking ART? | Years |  |
| 504 | ART regimen | 1. 1a 2. 1c 3. 1d 4. 1e 5. 1f 6. 2b 7. Others |  |
| 505 | Current or past opportunistic infection with in past 6 months | 1. No 2. Yes |  |
| 506 | If Q505 answer is yes which infection have    **(More than one answer is possible )** | 1. TB 2. Pneumonia 3. Oral thrush 4. Zoster 5. Diarrhea (acute/chronic) |  |
| 507 | Did you get support from any organization rather than medication | 1. Yes 2. No |  |
| 508 | If yes to “Q507 “ type of support  ***(Multiple answers are possible)*** | 1. Money 2. Food 3. Loan 4. Household Equipment or livestock |  |
| 509 | Did you get dietary counseling from health professionals | 1 Yes  2 No |  |
| 510 | Is their other person living with HIV on ART in your family? | 1. Yes 2. No |  |
| 511 | If yes to Q510 whom?  (More than one answer is possible) | 1. Spouse 2. My child 3. Parent 4. Other relative |  |
| 512 | Current physical condition | 1. Working 2. Ambulatory 3. Bed ridden |  |

**Part VI: Behavioral factors related questions**

| 601 | Do you smoke cigarette? | | 1. Yes 2. No | |  | |
| --- | --- | --- | --- | --- | --- | --- |
| 602 | Do you drink local or any kind of alcohol? | | 1. Yes 2. No | |  |  |
| 603 | During the past 30 days on how many days  did you have at least one drinking containing  alcohol | | 1. One 2. Two 3. Three and above | |  |  |
| 604 | Do you use khat | | 1 Yes  2 No | |  |  |
| 604 | Did you take all your medicines during the last 30 days? | | 1. Yes 2. No | |  |  |
| 605 | If Q604 is No, how many doses did you miss? | | doses | |  |  |
| 606 | What were the reasons you remember that forced you to miss your medicine? |  | | Yes | No |  |
|  |  | Had no food to take with medication | | 1 | 2 |  |
|  |  | I was away from home | | 1 | 2 |  |
|  |  | I was busy with other things | | 1 | 2 |  |
|  |  | I started Traditional medicine | | 1 | 2 |  |
|  |  | Had too many pills to take | | 1 | 2 |  |
|  |  | Wanted to avoid side effects | | 1 | 2 |  |
|  |  | Felt sick or ill | | 1 | 2 |  |
|  |  | Felt healthy/good | | 1 | 2 |  |
|  |  | Had problems taking pills at specified times | | 1 | 2 |  |
|  |  | Ran out of pills | | 1 | 2 |  |

**Part VI Anthropometric measurements**

| 701 | Weight (in Kg) | ____________ kg |
| --- | --- | --- |
| 702 | Height (in centimeter) | ____________ cm |

**Thank You very Much!!!**

**Name of the Data collector: ______________________________**

**Date: _____________________ Signature: _________________**

**Name of the Supervisor: _________________________**

**Date: _____________________ Signature: _______________**
